# Young Adults’ Selection and Use of Dependent Coverage under the Affordable Care Act

**DOI:** 10.3389/fpubh.2018.00003

**Published:** 2018-01-31

**Authors:** Weiwei Chen

**Affiliations:** ^1^Health Policy and Management, Florida International University, Miami, FL, United States

**Keywords:** young adults, dependent coverage, crowd out, insurance duration, Affordable Care Act

## Abstract

The dependent coverage expansion under the Affordable Care Act (ACA) required health insurance policies that cover dependents to offer coverage for policyholder’ children up to age 26. It has been well documented that the provision successfully reduced the uninsured rate among the young adults. However, less is known about whether dependent coverage crowded out other insurance types and whether young adults used dependent coverage as a fill-in-the-gap short-term option. Using data from the Survey of Income and Program Participation 2008 Panel, the paper assesses dependent coverage uptake and duration before and after the ACA provision among young adults aged 19–26 versus those aged 27–30. Regressions for additional coverage outcomes were also performed to estimate the crowd-out rate. It was found that the ACA provision had a significant positive impact on dependent coverage uptake and duration. The estimated crowd-out rate ranges from 27 to 42%, depending on the definition. Most dependent coverage enrollees used the coverage for 1 or 2 years. Differences in dependent coverage uptake and duration remained among racial groups. Less healthy individuals were also less likely to make use of dependent coverage.

## Introduction

The dependent coverage expansion, as one of the earliest and most popular provisions in the Affordable Care Act (ACA), has been the focus of considerable research in recent years. The expansion, implemented in September 2010, required all plans and issuers that offer dependent coverage to cover enrolled members’ children until age 26, regardless of living situation, marital or student status, or financial dependence. Although 37 states had enacted related state laws that extended the age limit before ACA (1), the effects of those state laws on young adults’ uninsured rate were found to be limited (2). The ACA’s dependent provision, however, was shown to be much more successful in increasing insurance coverage and improving other health care access measures. It is estimated that the insurance coverage rate of young adults (aged 19–25) increased by 3–5% points (3–6) and 2.3 million young adults (aged 19–25) gained dependent coverage since 2010 through October 2013 (7). A large number of studies also revealed the impact of ACA on health care utilization among young adults. The evidence of the findings is mixed, with some studies suggesting no impact whereas others found increases in emergency room visits, hospital stays, and outpatient visits (8–16).

Compared to the existing research mentioned above, relatively less attention has been paid to young adults’ insurance choices with the additional coverage option added to their choice set. Particularly, would young adults seek dependent coverage instead of their own policies provided by employers, i.e., dependent coverage crowded out employer-sponsored insurance (ESI) in own name? And did they use dependent coverage as a short-term option (to cover temporary gaps) or a longer-term option? These questions have important implications. Regarding payment distribution of health insurance and care cost, if the dependent coverage crowded out private coverage in own name, the financial burden of the insurance premium and health care cost would be transferred from dependent children and their employers to the parents and parents’ employers. The premium of family coverage would further increase because more people are covered under a family plan. When it comes to health care use, if young adults pick up dependent coverage only to cover short-term gaps in insurance transitions, it may result in frequent changes of insurance plans and unstable coverage. This may relate to the mixed results on health care usage found in earlier studies, since shorter duration and frequent plan change may lead to suboptimal use of health care.

The decision to take up dependent coverage can be trivial for young adults who were previously uninsured, it may not be so for others who already had some options. Consider someone who had an ESI in own name, or a plan from spouse, or a self-purchased plan, switching to parents’ plan would be largely due to financial reasons. Presumably, parents would pay the insurance premium for the family coverage after adult children were added. Compared with paying premium by oneself, it could be a big financial burden taken off from the shoulder. If the parents were already covering other dependents, the cost to add one more dependent would be little or zero. However, not all young adults like to be financially dependent on parents again or some might have concerns about the loss of privacy (their parents, as the primary policy holder, could review all medical claims unless adult children request not to release the information to parents). It is unclear whether individual characteristics would help predict the choice and use of dependent coverage.

This study aims to fill the gap by addressing two questions: (1) whether dependent coverage crowded out ESI in own name and other insurance coverage and (2) whether dependent coverage was used as a fill-in-the-gap short-term option or longer-term option. The evidence was drawn from longitudinal data of the Survey of Income and Program Participation (SIPP). A multivariate linear probability model was built in a difference-in-difference (DID) framework to examine the uptake of dependent coverage among young adults aged 19–26, relative to a control group of individuals aged 27–30, before and after the dependent provision. A Weibull hazard model was then built to analyze the duration of dependent coverage.

Earlier studies on insurance crowd-out arose after the initial Medicaid expansions of the 1987–1992 periods. The seminal work of Cutler and Gruber (17) examined the effect of Medicaid eligibility on Medicaid uptake and on private insurance coverage. Their crowd-out primarily referred to the reduction in private insurance relative to the growth in public insurance. They used a “simulated instrument” to address the endogeneity of Medicaid eligibility and found very high rates of crowd-out. Many of the subsequent studies adopted the simulated instrument strategy, with or without modifications, and applied it to different datasets to examine the same or different insurance expansions (18–24). They found crowd-out estimates are sensitive to model specification, dataset, and definition used. There were also studies that took a more straightforward approach by examining the trends in insurance coverage among the eligible group (25–27), or comparing the trends among both eligible and ineligible groups, which is similar to a DID approach (28–30). Some other studies used more direct measures of eligibility, such as Medicaid income eligibility threshold, to assess the impact of expansion (31, 32). In this study, the ACA expansion of dependent coverage has a simple eligibility rule—any adult children up to age 26 are eligible for the dependent coverage, I therefore chose the DID approach for the analysis.

To date, few studies have examined the crowd-out effect of dependent coverage expansion under ACA. In addition, little is known about what factors were associated with the duration of dependent coverage.

## Materials and Methods

### Data

The study used longitudinal data from SIPP 2008 Panel, waves 1–16. SIPP is a nationally representative longitudinal survey conducted by the US Census Bureau for evaluating the impact of federal, state, and local programs on income and economic well-being of individuals and households in the US. It samples from the US civilian non-institutionalized population and collects data for the same individual every 4 months on various topics on economic well-being, including health insurance. Interviews are conducted by personal visit and by decentralized telephone for all household members aged 15 or above. The 2008 Panel began in September 2008 and had originally interviewed over 52,000 households (33). The panel has been followed over a succession of staggered waves every 4 months until December 2013. In each wave, the respondents are asked to provide retrospective responses for the last 4 months. For the purpose of this study, SIPP dataset allows distinguishing private coverage under own name from dependent coverage and observing insurance status changes before and after leaving dependent coverage.

The study includes individuals aged 19–30 who participated in the 2008 Panel of SIPP. Totally, there are 138,484 person-month observations of 9,206 young adults who had dependent coverage and 639,730 person-month observations of 27,320 young adults who had no dependent coverage.

### Analysis

To illustrate the evolvement of young adults’ health insurance choices over the years, I estimated the monthly coverage rate by insurance type and by age group during May 2008 to November 2013 (the period for which retrospective responses were provided in SIPP interviews during September 2008 to December 2013), adjusting for personal weights. Four insurance types considered here include ESI in own name, parental-dependent coverage, public insurance (mostly Medicaid), and uninsurance. I also distinguished three age groups—19–22, 23–26, and 27–30 years old. The 27–30 years old were not affected by the ACA-dependent coverage expansion and were included as a comparison group. The 19–22 years old, though targeted by ACA, were more likely to be eligible for state-dependent coverage laws before the ACA provision. Therefore, they were separated from the 23 to 26 age group.

The study then examines insurance uptake and crowd-out using regression analysis. The crowd-out is defined in three ways. The first is the reduction in ESI in own name relative to the growth in dependent coverage; the second is the reduction in non-parental private insurance coverage (ESI in own name, spouse’ insurance, and individual purchased insurance) relative to the growth in dependent coverage; and the third is one minus (the change in uninsurance/the growth of dependent coverage). The three definitions gradually broaden the types of insurance considered for crowd-out, with the first one focusing on crowding out of ESI in own name, the second one on any private coverage, and the third one on uninsurance (what is left after any private or public coverage). If there was no crowd-out, there would be no reduction in ESI in own name or non-parental private coverage, and the uninsurance rate would decrease as much as the dependent coverage increased. Therefore, all three measures would be 0 in this case.

A linear probability model in a DID framework is built (as below) to examine the factors associated with insurance uptake. The coefficients representing the marginal effects would then be used directly for crowd-out estimation. Four coverage outcomes (dependent coverage, ESI in own name, any non-parental private coverage, or uninsurance) are considered for deriving crowd-out estimates, based on the three definitions:

(1) $$\begin{array}{l} Y_{\mathrm{its}}=\alpha +\beta_{1}{\text{ACA\_target}}_{it}+\beta_{2}{\text{ACA\_target}}_{it}\\ \;\times {\text{ACA\_effective}}_{t}+\beta_{3}{\text{State\_target}}_{its}+\beta_{4}{\text{State\_effective}}_{st}\\ \;+\beta_{5}{\text{State\_target}}_{its}\times {\text{State\_effective}}_{st}+X_{its}\gamma +\delta_{t}+\mu_{s}+\varepsilon_{its}. \end{array}$$

In Eq. 1, *Y_its_* denotes the probability that individual *i* of state *s* had a particular type of insurance coverage (dependent coverage, ESI in own name, any non-parental private coverage, or uninsurance) in month *t*. ACA_target*_it_* represents whether individual *i* belonged to the ACA targeted age group (≤26 years old) in month *t*. ACA_effective*_t_* indicates whether the ACA provision was effective in month *t* (=1 after September 2010; 0 otherwise). The interaction term ACA_target*_it_* × ACA_effective*_t_* is, therefore, the treatment indicator of the ACA. To control for impacts of state laws that extended dependent coverage before ACA, the model also introduced state policy variables, with State_target*_ist_* indicating whether individual *i* from state *s* was eligible for dependent coverage under state law in month *t*, and State_effective*_st_* denoting whether the law of state *s* was effective in month *t*. State_target*_ist_* was assigned based on dependent child’s residential state, age, marital status, whether they lived in the same state as their parents, and student status. State_effective*_st_* was assigned based on the year the state law became effective. Since our sample started in 2008, states that implemented their laws in or before 2008 had State_effective*_st_* equal to 1 in all months. States that implemented the law in 2009 had State_effective*_st_* equal to 1 from January 2009 onwards. There were 37 states that expanded dependent coverage by August 20, 2009 (1). The interaction term of State_target*_ist_* and State_effective*_st_* indicates the effect of state laws on dependent coverage. *X_its_* represents a vector of individual characteristics, including age (in years), gender, race, marital status, student status, education level (no high school diploma as the reference group, high school diploma only, some college, and college degree or above), self-reported health status (=1 if it is less than excellent; =0 if excellent), employment status (employed full-time, employed part-time, or unemployed), and income (in income-to-poverty ratio and its squared term). δ*_t_* includes fixed effects of each year-month and μ*_s_* controls for fixed effects by state. Lastly, ε*_its_* represents the error term. The standard errors were clustered at the individual level in all regressions.

Noticeably, the health measure was not available in all but only some waves which had topical modules covering health care utilization and medical expenses (wave 4, 7, and 10 covering interview months of September to December in 2009, 2010, and 2011, respectively). In this study, the self-reported health status from waves 4 to 7 were adopted. For months before wave 7, wave 4 health measure was applied to each month. For months during or after wave 7, wave 7 health measure was applied to each month. Wave 10 health measure was not used since it reflected health status 1 year after the ACA-dependent coverage expansion, which might be affected by the dependent coverage status after ACA.

The regression was first performed for dependent coverage to examine the factors associated with the uptake. It was then repeated for each of the other coverage outcomes. The evidence of crowd-out would be drawn from coefficient estimates of ACA’s impact on the related types of insurance coverage.

Lastly, the study examined the duration of dependent coverage among those ever enrolled in parent’ insurance during May 2008 to November 2013. The duration is defined as the number of continuous months covered under dependent coverage. For those who had multiple spells of dependent coverage (26% of all individuals who ever had dependent coverage), all spells were considered.

I used a Weibull model in the proportional hazard (PH) form for the duration analysis. The failure, in this case, refers to exit of dependent coverage. An alternative model—Cox PH model—was not chosen, because the PH-assumption test suggested the assumption was violated (global test *p*-value = 0.01). The model was built in a DID framework with the same variables as in model (1), except the dependent variable is now the duration of dependent coverage. The problems of potential right-censored and left-truncated observations in the survey have been taken into account in the duration model. The survival curve based on the Weibull regression, which shows the likelihood of remaining under dependent coverage in each duration month, is also presented. The standard errors were also clustered at the individual level.

For various types of non-responses, SIPP deals with it by weighting adjustments or imputation. All estimations in this study were adjusted using personal weights to be nationally representative. For the duration analysis, personal weights at the first month of the duration spell were used.

## Results

### Trends in Insurance Coverage by Insurance Type

**Figure 1:**
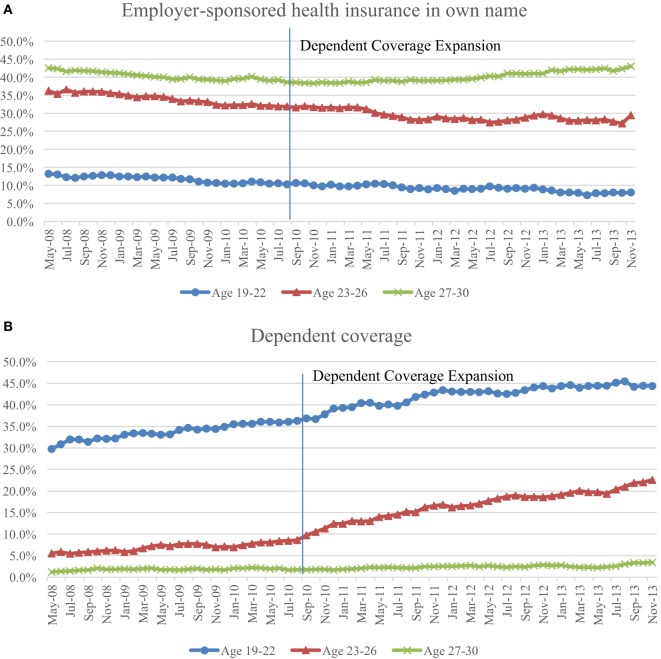

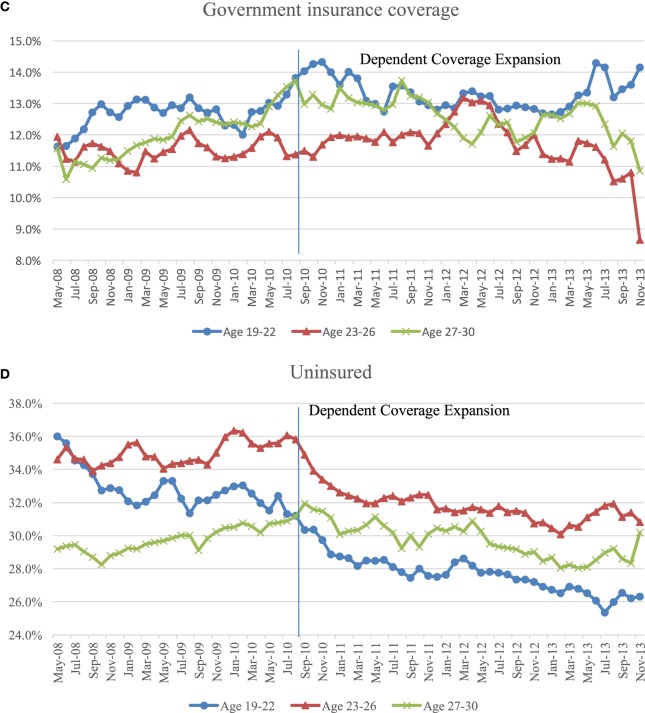
Insurance coverage dynamics among young adults aged 19–22, 23–26, and 27–30 during May 2008 to November 2013. **(A)** Percentages of individuals who had employer-sponsored health insurance in own name in each of the age groups. **(B)** Percentages of individuals who had dependent coverage through parents in each of the age groups. **(C)** Percentages of individuals who had government insurance in each of the age groups. **(D)** Percentages of individuals who were uninsured in each of the age groups.

Figure 1 presents the changes of insurance coverage rate by insurance type among three age groups (19–22, 23–26, and 27–30 years old). The insurance types reflect four common options faced by young adults—ESI in own name, parental-dependent coverage, government insurance coverage (primarily Medicaid), and uninsurance.

The rate of ESI in own name showed a declining trend among 19–22 and 23–26 age groups during the whole sample period (Figure 1A). The 27–30 age group, however, presented a slowly decreasing trend before the end of 2010 and a slowly increasing trend afterwards. This pattern of the 27–30 age group seems to reflect the trend of the US economy during the 2007–2009 recession and the recovery since 2010. On the contrary, the younger groups did not turn the downward curve up, indicating that the ACA-dependent provision might relate to the decline after 2010.

The dependent coverage rate, not surprisingly, went up over the years and appeared to grow at a faster pace after the expansion in September 2010, especially among those aged 23–26 (Figure 1B). As expected, no noticeable change was observed among the 27–30 age group. Combining the upward trend of dependent coverage rate and the downward trend of the rate of ESI in own name, it seems to suggest there was a crowd-out of ESI coverage in own name by the dependent coverage.

Government insurance coverage rate (Figure 1C), though appeared to fluctuate a lot, were within a narrow range of 11–14% for most of the sample years. In the end of 2013, there seemed to be an increase in the 19–22 age group and a quick decline in the older age groups. The overall trends, however, were less clear compared to other insurance types across the three age groups over the sample period.

Figure 1D depicts the trends of uninsured rate among the three age groups. After September 2010, all groups showed overall declining trends, with the 23–26 age group decreased from over 34% to nearly 30% and the 19–22 age group decreased from over 30% to around 26%. The 27–30 age group showed relatively less reduction and reached about 28% in 2013. These numbers agree with the post-ACA estimates of uninsured rate among all young adults ages 19–29, which range from slightly above 20 to 30% based on reports from multiple sources (34–37).

### Descriptive Statistics and Characteristics Associated with the Uptake of Dependent Coverage

Table 1 provides descriptive statistics of variables used in this study by dependent coverage status. Significant differences were observed in all characteristics between the two groups. For one of the race/ethnicity categories (other races), the two groups were close and only statistically different at 10% level. A higher proportion of person-month observations with dependent coverage were White, with student status, and had some college education. A lower proportion of them were married and had a full-time job. Those with dependent coverage also tended to be younger and had higher income than those without dependent coverage. The self-reported health status measure showed that less than half of the dependent coverage observations had less-than-excellent health status while the proportion for the non-dependent coverage observations was over 65%. There was also a high proportion of dependent coverage observations that were eligible for expanded dependent coverage under state law. The rate for non-dependent coverage observation was 33%, which was less than half of the rate for dependent coverage observations.

**Table 1:** Characteristics of young adults aged 19–30 with or without dependent coverage, May 2008 to November 2013.

	Dependent coverage		Non-dependent coverage		Dep = non-dep^a^
	Mean	SD	Mean	SD	
Female	0.498	0.500	0.509	0.500	***
Race					
White	0.725	0.446	0.595	0.491	***
Black	0.098	0.298	0.134	0.340	***
Hispanic	0.089	0.285	0.183	0.387	***
Asian	0.045	0.208	0.047	0.212	***
Other races	0.042	0.201	0.041	0.199	*
Married	0.035	0.184	0.303	0.460	***
Age	21.342	2.299	25.027	3.400	***
Student	0.601	0.490	0.211	0.408	***
Bad health: self-reported health status less than excellent	0.495	0.500	0.652	0.476	***
Employment status					
No job	0.428	0.495	0.313	0.464	***
Full-time job	0.530	0.499	0.655	0.475	***
Part-time job	0.042	0.201	0.032	0.175	***
Education level					
No high school diploma	0.031	0.172	0.108	0.311	***
High school graduate	0.246	0.431	0.312	0.463	***
Some college	0.598	0.490	0.365	0.481	***
College degree or above	0.125	0.331	0.214	0.410	***
Income (income-to-poverty ratio)	4.623	3.839	2.940	2.898	***
Eligibility under state law	0.703	0.457	0.333	0.471	***

Observations^b^	138,484		639,730		

^a^The last column reports t-test results for differences between those with or without dependent coverage. *** and * indicate 0.01 and 0.10 significance levels, respectively.

*^b^Observation unit is person-month. Bad health and income had fewer observations than other variables due to missing values (bad health: self-reported, dependent coverage: 114,294; bad health: self-reported, non-dependent coverage: 514,764; income, dependent coverage: 138,480; income, non-dependent coverage: 639,694)*.

### Result for Uptake of Dependent Coverage

A linear probability model examining how the policy variables and other individual characteristics were associated with young adults’ choices of dependent coverage is presented in Table 2. The likelihood of dependent coverage uptake among young adults under age 26 increased 7.4 percentage points after the ACA provision. The effect of state policies was not significant. Among the individual characteristics, all non-White races were less likely to take up dependent coverage than the Whites. People married, older, or with full-time jobs were less likely to enroll, whereas people with student status were more likely to enroll. It appears that individuals in less-than-excellent health were also less likely to take up dependent coverage than those reported better health. The education levels were positively associated with the uptake, though the effect of a college degree was not significant. For income, both the linear term and the square term were significant, meaning there was a non-linear effect. And the effect would be positive at first and decrease as income increases.

**Table 2:** Linear probability model examining dependent coverage uptake among young adults aged 19–30, May 2008 to November 2013.

	Coefficient	SE
Affordable Care Act (ACA)_target	−0.132***	(0.006)
ACA_target_effective	0.074***	(0.005)
State_target	0.079***	(0.012)
State_effective	0.009	(0.007)
State_target_effective	−0.009	(0.011)
Female	0.007*	(0.004)
Race (White as ref.)		
Black	−0.077***	(0.006)
Hispanic	−0.060***	(0.006)
Asian	−0.045***	(0.010)
Other races	−0.037***	(0.011)
Married	−0.056***	(0.004)
Age (in years)	−0.040***	(0.001)
Student	0.134***	(0.004)
Bad health	−0.030***	(0.004)
Employment status (no job as ref.)		
Full-time job	−0.052***	(0.004)
Part-time job	−0.005	(0.005)
Education level (no high school diploma as ref.)		
High school diploma only	0.013**	(0.006)
Some college	0.075***	(0.006)
College degree or above	0.003	(0.007)
Income (in income-to-poverty ratio, IPR)		
IPR	0.040***	(0.001)
IPR^2^	−0.001***	(0.000)

*R*-square	0.312	

*p < 0.1.

**p < 0.05.

***p < 0.01.

*The table reports coefficients and SEs (in parenthesis) of a linear probability model, with dependent coverage uptake as the dependent variable. The model included individuals aged 19–30 during May 2008 to November 2013, with 629,038 individual-month observations. The regression also included fixed effects of states and year-month. Estimations were adjusted using personal weights to be nationally representative. The SEs were clustered at individual level*.

### Results for Crowd-Out

To estimate the crowd-out, separate regressions were performed for three additional coverage outcomes (ESI in own name, non-parental-private insurance, and uninsurance). The full results are presented in Appendix Table A1 in Supplementary Material. In addition to the significant increase in dependent coverage uptake (7.4 percentage points) associated with ACA, there were also significant decreases in the uptake of ESI in own name, non-parental insurance, and uninsurance (range from 2.0 to 4.3 percentage points) associated with ACA. Based on the coefficient estimates, the crowd-out is calculated to be 27%, 35%, and 42% according to the three definitions, respectively (Table 3). It implies that every percentage point increase in dependent coverage uptake would result in 0.27 percentage point decrease in ESI in own name; 0.35 percentage point decrease in any non-parental private coverage; and 0.58 percentage point (=1–0.42) decrease in uninsurance (would be 1 percentage point decrease in uninsurance if no crowd-out).

**Table 3:** Effect of Affordable Care Act (ACA)-dependent coverage expansion on insurance coverage.

Dependent coverage	Employer-sponsored insurance in own name	Non-parental private insurance	Uninsured	Crowd 1	Crowd 2	Crowd 3
0.074***	−0.020***	−0.026***	−0.043***	0.27	0.35	0.42
(0.005)	(0.008)	(0.008)	(0.008)			

***p < 0.01.

*The reported are estimates of ACA’s effect on each of the coverage outcomes from four separate regressions. SEs are in parentheses. Crowds 1, 2, and 3 represent the derived crowd-out rates based on three definitions*.

### Results for Coverage Duration

**Figure 2:**
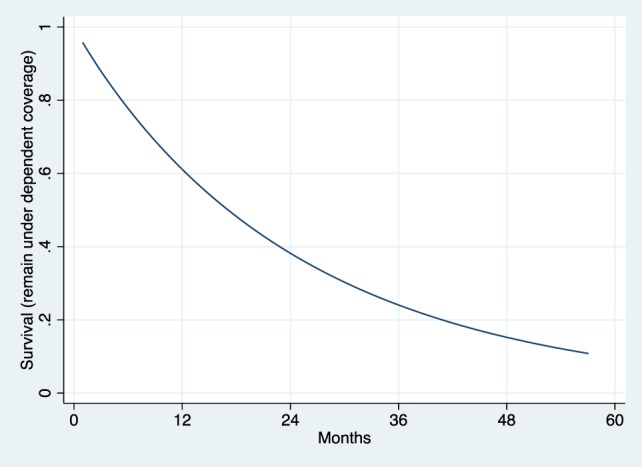
Weibull survival curve of dependent coverage retention.

The Weibull regression in PH form was used for the duration analysis. The survival curve (Figure 2) depicts the likelihood of remaining under dependent coverage by month after enrollment. Among those ever had dependent coverage during May 2008 to November 2013, over 60% remained covered at 12 months after enrollment. The likelihood went below 40% at 24 months. By 36 months, the likelihood of remaining under dependent coverage was around 25%. Therefore, over half of young adults dropped the coverage within 2 years. It implies that they tended to use the dependent coverage as a short-term option.

The Weibull regression result predicting the dependent coverage duration is shown in Table 4. The reported hazard ratios represent the hazard of losing dependent coverage. An estimate of value greater than 1 means increased hazard (therefore shorter duration) and a value less than 1 implies reduced hazard (therefore longer duration).

**Table 4:** Weibull regression for the duration of dependent coverage among young adults aged 19–30 who had ever covered by parent’s plan, May 2008 to November 2013.

	Haz. ratio	SE
Affordable Care Act (ACA)_target	1.833***	(0.178)
ACA_target_effective	0.649***	(0.060)
State_target	0.789	(0.138)
State_effective	1.001	(0.173)
State_target_effective	1.026	(0.181)
Female	0.927**	(0.031)
Race (White as ref.)		
Black	1.379***	(0.071)
Hispanic	1.189***	(0.072)
Asian	1.115	(0.087)
Other races	1.071	(0.093)
Married	1.735***	(0.117)
Age (in years)	1.171***	(0.011)
Student	0.787***	(0.029)
Bad health	1.063*	(0.035)
Employment status (no job as ref.)		
Full-time job	1.138***	(0.039)
Part-time job	1.056	(0.079)
Education level (no high school diploma as ref.)		
High school diploma only	0.998	(0.097)
Some college	0.785**	(0.075)
College degree or above	1.023	(0.102)
Income (in income-to-poverty ratio, IPR)		
IPR	0.899***	(0.007)
IPR^2^	1.003***	(0.000)

Aux *p*	0.97	
*N*	95,522	

*p < 0.1.

**p < 0.05.

***p < 0.01.

*The table reports hazard ratios and SEs (in parenthesis) of a duration analysis using Weibull model in the proportional hazard form. The model included individuals aged 19–30 who ever had dependent coverage during May 2008 to November 2013. The regression also included fixed effects of states and year-month. Estimations were adjusted using personal weights (at the first month of dependent coverage) to be nationally representative. The SEs were clustered at individual level. Aux p represents the shape parameter *p* of the Weibull model*.

The results reveal a significant effect of ACA on the duration of dependent coverage. The term ACA_effective_target had a hazard ratio of 0.65, meaning the hazard of losing the dependent coverage was reduced by 35% (=100–65%) for those up to 26 years old after the ACA was implemented. The effect of state laws on duration was not significant.

Among individual characteristics, race/ethnicity groups were again significantly associated with dependent coverage retention or duration. Black young adults, once enrolled, tended to stay shorter under dependent coverage than the Whites, with the hazard of leaving dependent coverage 38% higher than the White. Similarly, the hazard among Hispanic young adults was 19% higher than the White.

Young adults who were married, or older, or had a full-time job were also more likely to leave dependent coverage and had a shorter duration of coverage, compared to their counterparts. Those with student status were more likely to stay under coverage longer. Compared with males, females were slightly less likely to leave dependent coverage. Young adults who self-reported to have less-than-excellent health had slightly higher hazard than individuals with excellent health. Lastly, income had a significant non-linear effect on duration.

## Discussion

This paper assesses young adults’ insurance selection and coverage dynamics using longitudinal survey data from SIPP. The result of ACA’s positive impact on insurance coverage is consistent with previous literature (16). The estimate of 7.4 percentage points increase in dependent coverage is in line with earlier findings (3).

The study also provides new evidence on crowd-out. It has been observed in an earlier study (38) and also found in this study that there was a drop in the rate of ESI in own name and an increase in the dependent coverage simultaneously after the dependent coverage provision in 2010. Cantor et al. (3) used data of Current Population Survey 2005–2011 and found no strong evidence of dependent coverage crowding out private self or spouse insurance (only one of the four models produced significant estimates). However, by comparing their estimated decreases in uninsured rates with the increases in dependent coverage rates, there seem to be considerable crowd-out in terms of uninsurance changes. Based on regression estimates in this study, I found crowd-out rate ranging from 27% to 42%, depending on the definition. Notice that before January 1^st^ 2014, the law allowed insurers to deny dependent coverage if the young adult had another offer of employer-based coverage through his or her own job (39). The crowd-out could be greater when the restrictions no longer held beginning in 2014. This may further increase the premium of private family coverage as the average family size increases.

It was also found that most young adults tended to use their dependent coverage for 1 or 2 years. By the end of a 2-year period, less than 40% still kept their dependent coverage. The duration did not appear to be longer for those were less healthy. Given the earlier studies which found no significant increase in some health services use, transitioning in and out of dependent coverage in 1 or 2 years may lead to instable insurance coverage and ultimately discourage health care use.

Among the other factors, it is noticeable that racial differences were found in both the uptake and duration models, with Blacks and Hispanics less likely to enroll or stay long under dependent coverage. The estimates of individual characteristics, in line with previous research (40–41), suggest that White Americans and young adults from higher-income families benefited more from the provision than their counterparts. In addition, the result that less healthy individuals were less likely to take up dependent coverage may imply that the high risk was among non-parental insurance plans.

### Limitations

There are several limitations of this study. First, since the health measure was not available for each survey month, measures from waves 4 to 7 (covering interviewing months September to December in 2009 to 2010, respectively) were used. Second, some caveats for the survey data might also apply. SIPP data might include recall bias (due to the 4-month recall period used in the survey) and seam bias (known as respondents’ tendency to report changes across the “seam” between two successive survey administrations rather than within a single interview) (42, 43). Third, the variable State_target*_ist_* used to control the effect of states laws before ACA is a rough measure of state-dependent coverage eligibility. It was based on residential state, age, marital status, student status, and whether the dependent child lived in the same state as his or her parents. There were other restrictions or exemptions made by the states but not included here. For example, state laws did not apply to large self-insured group plans. Since SIPP does not provide information about whether a plan is self-insured group plan or not, State_target*_ist_* does not take into account whether parental insurance was subject to the state law. However, the effect of state laws on insurance coverage was found to be very limited compared to the impact of ACA (2). And my results also confirmed the previous findings.

## Conclusion

Despite the limitations, the results would help us better understand young adults’ selection and use of dependent coverage among all other options. The uptake of dependent coverage might have crowded out young adults’ own ESI and other insurance policies. Young adults tended to use dependent coverage as a temporary option for 1–2 years. Differences in dependent coverage uptake and duration remained among racial groups. Less healthy individuals were also less likely to make use of dependent coverage.

As to the national goal of expanding insurance coverage, what the country has experienced so far provides valuable evidence for future policy making. The crowd-out may further increase the premium of family coverage as more adult children join parents’ plan. Additional attention also needs to be paid to differences in uptake and duration by race and by health status.

## Ethics Statement

This study is not considered as human subjects’ research and does not require IRB review, because all the data used in this study were from a publicly available survey and were de-identified.

## Author Contributions

The author is fully responsible for the design, analysis, and writing of the manuscript.

## Conflict of Interest Statement

The author declares that the research was conducted in the absence of any commercial or financial relationships that could be construed as a potential conflict of interest.
