# Supplementary material for: Young Adults’ Selection and Use of Dependent Coverage under the Affordable Care Act
Source: Front Public Health. 2018 Jan 31;6:3. doi: 10.3389/fpubh.2018.00003 (PMC5797739; doi:10.3389/fpubh.2018.00003)
Supplement: Supplementary file 1 [file Table_1.PDF]

Appendix

Table A1: Linear Probability Model Examining Insurance Coverage among Young Adults Aged 19 to 30, May 2008 -Nov 2013

	Dependent Coverage		ESI in own name		Non-parental- private insurance		Uninsured	
	Coef.	SE	Coef.	SE	Coef.	SE	Coef.	SE
ACA_target	-0.132***	(0.006)	0.038***	(0.008)	0.047***	(0.008)	0.086***	(0.008)
ACA_target_effective	0.074***	(0.005)	-0.020***	(0.008)	-0.026***	(0.008)	-0.043***	(0.008)
State_target	0.079***	(0.012)	-0.046***	(0.014)	-0.033**	(0.014)	-0.013	(0.014)
State_effective	0.009	(0.007)	-0.001	(0.010)	0.005	(0.010)	-0.013	(0.010)
State_target_effective	-0.009	(0.011)	0.004	(0.013)	-0.007	(0.013)	-0.005	(0.013)
Female	0.007*	(0.004)	-0.038***	(0.005)	-0.003	(0.005)	-0.071***	(0.005)
Race (White as ref.)								
Black	-0.077***	(0.006)	0.006	(0.008)	-0.049***	(0.008)	0.045***	(0.009)
Hispanic	-0.060***	(0.006)	-0.021***	(0.008)	-0.089***	(0.009)	0.158***	(0.009)
Asian	-0.045***	(0.010)	-0.019	(0.012)	-0.005	(0.013)	0.077***	(0.013)
Other races	-0.037***	(0.011)	0.001	(0.013)	-0.029**	(0.014)	0.026*	(0.014)
Married	-0.056***	(0.004)	-0.033***	(0.007)	0.146***	(0.007)	-0.083***	(0.007)
Age (in yrs)	-0.040***	(0.001)	0.023***	(0.001)	0.026***	(0.001)	0.011***	(0.001)
Student	0.134***	(0.004)	-0.067***	(0.005)	-0.026***	(0.005)	-0.082***	(0.005)
Bad health	-0.030***	(0.004)	-0.003	(0.005)	-0.022***	(0.005)	0.024***	(0.005)
Employment status (no job as ref.)								
Full-time job	-0.052***	(0.004)	0.261***	(0.004)	0.194***	(0.005)	-0.033***	(0.005)
Part-time job	-0.005	(0.005)	0.038***	(0.004)	0.014***	(0.006)	0.060***	(0.007)
Education level (no high school diploma as ref.)								
High school diploma only	0.013**	(0.006)	0.078***	(0.007)	0.110***	(0.008)	-0.083***	(0.011)
Some college	0.075***	(0.006)	0.097***	(0.008)	0.156***	(0.009)	-0.142***	(0.011)
College degree or above	0.003	(0.007)	0.259***	(0.010)	0.364***	(0.010)	-0.231***	(0.012)
Income (in income-to-poverty ratio, IPR)								
IPR	0.040***	(0.001)	0.025***	(0.002)	0.030***	(0.002)	-0.046***	(0.002)
IPR^2	-0.001***	(0.000)	-0.001***	(0.000)	-0.001***	(0.000)	0.001***	(0.000)
R-square	0.312		0.260		0.299		0.178	

Note. *** p<.01, ** p<.05, * p<0.1. The table reports coefficients and standard errors (in parenthesis) of four linear probability models. The dependent variable is dependent coverage, ESI in own name, non-parental-private insurance, and uninsured, respectively. The model included individuals aged 19 to 30 during May 2008 to Nov 2013, with 629,038 individual-month observations. The regression also included fixed effects of states and year-month. Estimations were adjusted using personal weights to be nationally representative. The standard errors were clustered at individual level.
